# Audio motor training improves mobility and spatial cognition in visually impaired children

**DOI:** 10.1038/s41598-019-39981-x

**Published:** 2019-03-01

**Authors:** Giulia Cappagli, Sara Finocchietti, Elena Cocchi, Giuseppina Giammari, Roberta Zumiani, Anna Vera Cuppone, Gabriel Baud-Bovy, Monica Gori

**Affiliations:** 1 Unit for Visually Impaired People, Center for Human Technologies, Fondazione Istituto Italiano di Tecnologia, Genova, Italy; 2 Istituto David Chiossone per Ciechi ed ipovedenti ONLUS, Genova, Italy; 3 Centro regionale per l’ipovisione in età evolutiva, IRCCS Scientific Institute “E. Medea”, Bosisio Parini, Lecco, Italy; 4 IRIFOR del Trentino, Trento, Italy; 5 RBCS Robotics, Brain and Cognitive Science department, Center for Human Technologies, Fondazione Istituto Italiano di Tecnologia, Genova, Italy; 6 Vita-Salute San Raffaele University & Unit of Experimental Psychology, Division of Neuroscience, San Raffaele Scientific Institute, Milan, Italy

## Abstract

Since it has been demonstrated that spatial cognition can be affected in visually impaired children, training strategies that exploit the plasticity of the human brain should be early adopted. Here we developed and tested a new training protocol based on the reinforcement of audio-motor associations and thus supporting spatial development in visually impaired children. The study involved forty-four visually impaired children aged 6–17 years old assigned to an experimental (ABBI training) or a control (classical training) rehabilitation conditions. The experimental training group followed an intensive but entertaining rehabilitation for twelve weeks during which they performed ad-hoc developed audio-spatial exercises with the Audio Bracelet for Blind Interaction (ABBI). A battery of spatial tests administered before and after the training indicated that children significantly improved in almost all the spatial aspects considered, while the control group didn’t show any improvement. These results confirm that perceptual development in the case of blindness can be enhanced with naturally associated auditory feedbacks to body movements. Therefore the early introduction of a tailored audio-motor training could potentially prevent spatial developmental delays in visually impaired children.

## Introduction

Visually impaired children tend to manifest impairments in the development of spatial abilities, specifically in auditory and proprioceptive spatial localization^1,2^, haptic orientation discrimination^3^ and reach on sound^4^. They also show developmental delays in several motor skills^5^ and slower walking speed associated with prolonged duration of stance phase^6^.

Trainings commonly adopted in the case of visual disability are mostly unimodal, that is to say they tend to enhance the residual visual information through intensive and repetitive visual activities^7^ or substitute the visual input with a vicarious (auditory or tactile) input through sensory substitution devices that transform the visual properties of a scene into sonorous or tactile stimuli^8–10^. Nonetheless the benefit of multimodal stimulation in enhancing perceptual functions and learning has been repeatedly demonstrated^11–13^ and it is linked to the fact that perceiving coherent cross-modal stimuli provides the basis for multisensory redundancy that helps to detect the amodal properties of events^14^. Therefore, the early adoption of training approaches based on multisensory training would increase and improve learning opportunities for visually impaired people.

Positive outcomes of multisensory stimulation have been demonstrated in the case of individuals with partial visual deficits, indicating that an audio-visual training can in fact facilitate long-lasting visuo-spatial functions^15–17^ and possibly produce long-term plastic changes^18^. These results suggest that enriched experience with crossmodal stimulation can reinforce brain potential to perceive the multisensory nature of events.

To date no studies assessed the potential of multisensory training in the case of individuals with complete vision loss, mainly because much of the effort has been put in the development of sensory substitutions devices (SSD) that aim at substituting the missing sense (i.e. vision) by conveying the information generally transmitted by the missing sense with a different sensory channel (i.e. tactile or auditory). While these devices can provide support for specific perceptual tasks in adults^10^, they have never been tested in children principally because their use might overwhelm children attentional resources and require extensive training^19^. Nonetheless innovative rehabilitation trainings should be addressed especially to children because cortical plasticity is maximal in the first period of life and thus the benefit deriving from effective rehabilitation trainings should be higher.

As regards the importance of multisensory training, one study from our group assessed the impact of a short multisensory audio-motor training on spatial abilities of blind adults^20^, demonstrating that it can improve the encoding of audio motion. However, although it has been indicated that multisensory-based training trainings should be adopted early in therapeutic care to facilitate the development of body awareness^21^, very few studies tested the possibility that a similar improvement can be obtained with visually impaired children. Also, some studies assessing the impact of multisensory training in the form of virtual reality environments on the development of spatial perception showed that such technologies combining audio and haptic features might support blind people in their anticipatory exploration and cognitive mapping of the unknown space^22–25^. Our group preliminarily tested the efficacy of a multisensory rehabilitation training on young visually deprived children showing the potential of combining information across senses during early childhood^26^. Nonetheless, the mentioned study was conducted on a small sample (n = 7) of children, preventing the possibility to claim more general and definitive conclusions about the efficacy of such an early therapeutic intervention and about the different impact of multisensory stimulation depending on visual impairment severity.

**Figure 1:**
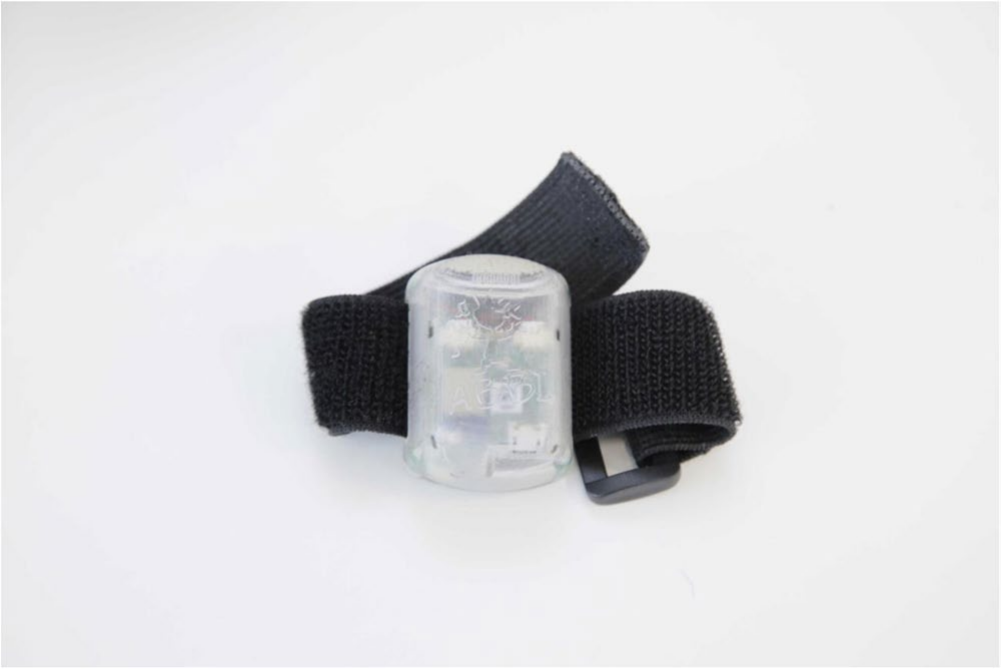
ABBI, the Audio Bracelet for Blind Interaction, is a small wearable custom-designed technology with integrated audio system, motion sensors, and a Bluetooth low energy module to communicate with a smartphone. The core idea behind ABBI is to use the auditory modality to convey spatial information about the movement of the person’s own body within the personal, peri-personal and extra-personal space.

For this reason, the present study aimed at investigating in a more rigorous manner the effects on spatial development of a new training based on multisensory stimulation in children across childhood. The training we propose uses a newly developed technology, the Audio Bracelet for Blind Interaction (ABBI), that produces an auditory feedback of body movements when positioned on a main effector such as the wrist in order to provide a sensorimotor signal similar to that used by the sighted child to construct a sense of space (Fig. 1, for additional information see^27^). As several reports indicate that sighted children typically acquire spatial competence by experiencing visuo-motor correspondences^28^, our approach could be used to align the spatial understanding between one’s own body and the external space through coupling auditory feedback with intentional motor actions. In fact, the audio movement conveys spatial information and allows the individual to build a representation of the movement in space in an intuitive and direct manner. This simple technology, coupled with a complete set of exercises, constitutes the ABBI training.

## Methods

### Participants

Visually impaired children aged 6–17 years old were enrolled by three different Italian training centers: Istituto Chiossone (IC), IRCCS Eugenio Medea (EM), and IRIFOR (IT). As required by the Italian Law, the study was separately approved by the relevant ethical committee of the region to which the training center belongs to, namely ASL3 Genovese for IC, IRCCS Eugenio Medea for EM, and APSS Trentino for IT. The study was carried out in accordance with the Helsinki Declaration guidelines. All parents and children, where requested by the Ethical committee, gave written informed consent in participating to the study.

**Figure 2:**
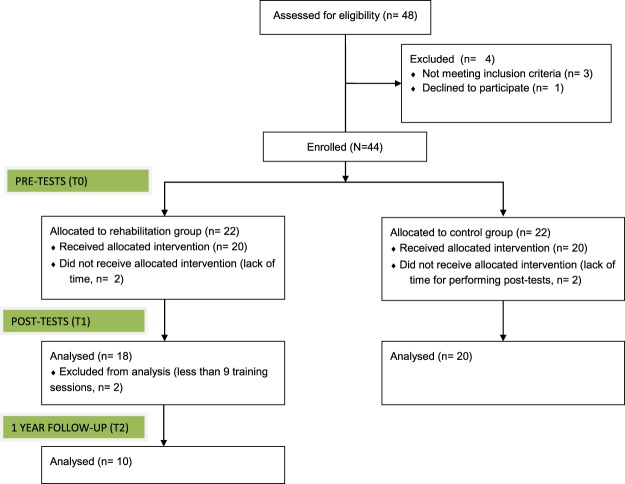
CONSORT diagram with participant flow.

Children were considered eligible for the study if they were completely blind or had residual visual acuity less than 1/10, had no other additional disability, and had an appropriate cognitive level for their age. The visual deficit was assessed according to the International Statistical Classification of Diseases and Related Health Problems (ICD) tenth revision^29^, according to which severe visual impairment (category 2) is related to visual acuity less than 0.5–1.3 LogMAR and complete blindness (category 3,4,5) is related to visual acuity less than 1.3 LogMAR to light perception. The cognitive level of all the visually impaired children was assessed with ‘The Reynell-Zinkin Scales: Developmental Scales for Young Visually Handicapped Children’ or the verbal scale of the ‘Wechsler Intelligence Scale for Children (WISC)’^30^ and considered appropriate for their participation in the study. Details of the visually impaired participants are indicated in Table 1. Forty-four children were initially enrolled in the study, of which thirty-eight were analyzed (for details, see Fig. 2).

**Table 1:** Clinical details of the participants.

Sex	Age	Pathology	Visual Acuity	Onset
**ABBI Intervention**				
F	6	Retinopathy of prematurity	Light perception	Congenital
M	6	Ocular albinism	1	Congenital
M	7	Neurofibromatosis type I	1.7	Acquired
M	8	Leber’s amaurosis	1	Congenital
F	8	Microphtalmia	1	Acquired
F	9	Retinopathy of prematurity	Light perception	Congenital
F	9	Retinopathy of prematurity	None	Congenital
F	9	Retinopathy of prematurity	Light perception	Congenital
M	10	Tuberculous meningitis	Light perception	Congenital
M	10	Optic nerve glioma	0.1	Congenital
F	11	Nystagmus	0.7	Acquired
F	12	Congenital cataract and microphtalmia	Light perception	Congenital
F	12	Retinopathy of prematurity	Light perception	Congenital
M	13	Optic nerve glioma	0.7	Acquired
F	13	Stargardt’s Macular Distrophy	1	Acquired
F	14	Leber’s amaurosis	Light perception	Congenital
F	14	Optic nerve atrophy	1	Congenital
F	14	Optic nerve atrophy	1.7	Acquired
F	15	Retinopathy of prematurity	None	Congenital
M	15	Retinopathy of prematurity	None	Congenital
F	16	Cones dystrophy	Light perception	Congenital
M	17	Leber’s amaurosis	Light perception	Congenital
**Classical Intervention**				
M	6	Retinal dystrophy	2	Acquired
M	6	Albinism	1.3	Congenital
M	7	Albinism	1	Congenital
M	7	Norrie syndrome	Light perception	Congenital
F	7	Complex ocular malformation	Light perception	Congenital
M	8	Retinal dystrophy	1.3	Congenital
M	8	Achromatopsia	1.3	Congenital
F	9	Congenital nystagmus	1	Congenital
F	9	Optic atrophy	Light perception	Congenital
F	9	Astrocytomas	1.3	Acquired
M	10	Retinal dystrophy	1.22	Acquired
M	10	BiAcquiredral retinoblastoma	2	Congenital
F	11	Achromatopsia	1	Congenital
F	11	Leber’s amaurosis	2	Congenital
M	11	Lyell syndrome	1.3	Acquired
F	12	Retinal dystrophy	1	Congenital
F	12	Optical nerves hypoplasia	1.7	Congenital
M	13	Retinal dystrophy	1.22	Congenital
F	13	Retinopathy of prematurity	1	Congenital
M	13	Optic atrophy	1	Acquired
M	15	Retinal dystrophy	1.22	Acquired
F	15	Maculopathy	1.3	Acquired

Twenty-two (14 females, mean age 11 +/− 3) children participated in the ABBI intervention, and twenty-two (10 females, mean age 10 +/− 3) children participated in the Classical intervention. The table shows the gender, the age at test, the principal diagnosis, the visual acuity expressed as LogMAR and the onset of visual disfunction.

### Procedure

The study consisted of three sessions: pre-evaluation, training, and post-evaluation session. Pre- and post- evaluation session lasted 60 minutes during which a battery of spatial and motor tests were performed. The training session lasted 12 weeks, during which children could be assigned to the ABBI training based on activities performed with the use of ABBI or to the classical training based on psychomotor lessons not necessarily involving sound localization activities. All children participating in the ABBI training (n = 22, mean visual acuity = 1.5 LogMAR) were enrolled in IC, while all children participating in the classical training (n = 22, mean visual acuity = 1.4 LogMAR) were recruited in EM and IT. The two groups were matched for age (see Table 1) and no significant differences exist among the two groups for residual visual acuity (t_(42)_ = 0.8 p = 0.42). Causes of visual impairment were balanced between groups according to the distinction between visual deficits of peripheral (ABBI training group n = 12, classical training group n = 14) vs central origin (ABBI training group n = 10, classical training group n = 8). Moreover, children in the two groups were balanced for what concerns onset of visual impairment (ABBI training group: 16 children with congenital and 6 children with acquired visual disability; Classical training group: 15 children with congenital and 7 children with acquired visual disability). All participants were naïve to the study hypothesis. One year after the post-evaluation session, ten children that participated in the ABBI training were re-tested in a follow-up session.

### The training

All children enrolled in the ABBI training performed dedicated training sessions with the use of ABBI. Specifically, each child performed weekly training sessions with a trained rehabilitator at IC for 45-minutes (9 hours over 12 weeks) and weekly training sessions with a relative at home for 5-hours (60 hours over 12 weeks) for a total of 69 hours. A variety of sounds were played during the training and these were selected depending on user’s preference^31^. Sounds could be pure tones (intermittent or continuous) or preselected playback sounds chosen by the child and stored in the device (e.g. elephant, mosquito, drums). The preselected sounds were equally informative for the exercises proposed^32,33^. The motivation of making these sounds customizable according to children’ preferences was to keep the usesr motivated and to let them enjoy the training session.

Furthermore, during the training sessions, children performed several spatial exercises during which ABBI could be worn both by the child or by the therapist/relative in order to make the training more entertaining. All spatial exercises were developed by researchers of IIT and therapists of IC and were meant to train the children’ ability to recognize and localize sounds in space according to different levels of difficulty:

- First level: recognize and localize simple sound movements, such as a straight motion flow performed along the horizontal or sagittal planes in the front peri-personal space;
- Second level: recognize and localize complex sound movements, such as a motion flow performed randomly in space in the front peri-personal space, e.g. composite geometrical and non-geometrical figures;
- Third level: recognize and localize simple and complex sound movements in the back peri-personal space;
- Fourth level: recognize and localize simple and complex sound movements in the front and back in the extra-personal space.

### Evaluation tests

In order to evaluate the effectiveness of the training, we developed a battery of tests to measure spatial and motor skills of children with visual disability.

These tests have been previously validated as effective tools for measuring spatial cognition and mobility in visually impaired with respect to sighted individuals^1,2,34^. Six tests were considered:

#### Auditory localization

The child listened to the sound produced by one of the twenty-three loudspeakers positioned in the horizontal plane in front of him and then used a cane to point to the loudspeaker that produced the sound. For each subject the mean error score in degrees was considered for analysis as the average of the difference in degrees between the physical and the indicated position.

#### Auditory bisection

The child listened to a sequence of three sounds presented successively by three of the twenty-three loudspeakers positioned in the horizontal plane in front of him and verbally reported whether the second sound was closer in space to the first or to the third one presented. For each subject the proportion of rightward responses was calculated by measuring the distance between loudspeakers.

#### Auditory distance

The child listened to two consecutive sounds produced from the twenty-three loudspeaker array positioned in front of him in the depth dimension and had to verbally report which of the two stimuli presented was closer in space to his own body. For each subject the proportion of trials where the probe was judged closer than the standard was computed for each probe position.

#### Auditory reaching

The child listened to a static sound (5 seconds) positioned in the space and reached the position of the sound. Three different positions were randomly reached three times, for a total of nine trials. Accuracy was calculated as the distance in centimeters between the real sound position and the actual reached position by the child.

#### Proprioceptive reaching

The child’s hand was passively guided by the experimenter from a fixed starting position towards a specific position of the space and then back to the starting position. The child was then asked to reproduce the whole movement trajectory. Three different positions were randomly reached three times (1 m, 1.7 m, 2.5 m), for a total of nine trials. Accuracy was calculated as the distance in centimeters between the real final position and the actual position reached by the child.

#### General mobility

This test was adopted and modified from the Timed up and go test^35^. The child stood up in the origin position with the feet aligned. The child was given a signal “ready, 1, 2, 3, and go.” On the go cue, the child was asked to walk until the experimenter touched his shoulder (indicating 3 m), turned around and walked back to the origin position. The test was repeated three times. For each repetition the time in seconds was recorded from the “go” cue to when the child went back to the origin position with a stopwatch.

### Data and Statistical Analysis

Based on meta-analysis of previous exercise-cognition studies in children^36^ we expected a medium effect size. Such an effect size could be statistically detected with a total sample size of 32 participants for a 1-tailed sample t-test (power 0.70, alpha 0.05).

Details on the data analysis of each evaluative test are indicated. In the auditory localization test, localization error was calculated for each participant as the difference (in centimeters) between the correct position and the indicated position for all trials. The average score was then converted in angular distance, namely from centimeters to degrees, for each group of participants, and the average error score in degrees was considered for analysis. We considered the correct position as the midpoint of each loudspeaker. The minimum possible error was 1.9 degrees (equal to 5 cm), equal to the distance between the midpoints of two next loudspeakers. Ambiguous situations (e.g. the participant pointed between two loudspeakers) were resolved by asking the participant to move slightly right or left the cane from the position previously indicated to better encode the answer.

For the auditory bisection and distance tests, the proportion of rightward responses was calculated for each speaker distance, and the data were plotted with a cumulative Gaussian distribution (error function). Following standard psychophysical procedure^37^, discrimination thresholds were taken to be the standard deviation of these distributions. Localization error in centimeters was converted in localization error in degrees.

For the auditory and proprioceptive reaching tests, the localization error was calculated for each participant as the average of the difference (in centimeters) between the correct position and the position reached for all trials. In the general mobility test, the time to complete the task has been averaged for each participant and then averaged for each group.

### Then the normality of distribution of each data test was verified by the Kolmogorov-Smirnov test

In order to evaluate the effects within groups, two-tailed t-tests assuming equal variances were performed between groups at baseline (T0) and post-training period (T1). Changes in the outcome measures were then calculated between baseline (T0) and post-training period (T1) in the ABBI training and classical training group (Δ_Α_ and Δ_C_), and between baseline (T0) and follow-up period (T2) in the ABBI training group (Δ_Α2_). In order to verify the effects of ABBI training vs classical training, the effects were compared by means of ANCOVA: in the linear models, the Δ scores were compared between groups and adjusted for the baseline score which was included as covariate^38–40^. As standardized effect size, Cohen’s *d* and 95% confidence intervals (CI) were also calculated. In order to verify the long-lasting effect of the ABBI training, two different group tests were performed. To evaluate the effects within groups, two-tailed t-tests assuming equal variances were performed at post-training (T1) and 1-year follow-up (T2). In addition, Wilcoxon signed rank tests were performed between (T1-T0) and (T2-T0). The threshold for statistical significance was set to p < 0.05.

## Results

The spatial performance of visually impaired children included in the training group significantly improved compared to the one included in the control group.

The spatial performance of the training group in the post-training session and a follow-up session performed one year after the formal end of the training suggests the presence of long-lasting effects due to the audio-motor training. Results are shown in Table 2.

**Table 2:** Score difference (Δ) after 12 weeks training (T1-T0).

	ABBI group (Δ_A_ = T1 − T0, N = 18)	Control group (Δ_C_ = T1 − T0, N = 20)	ABBI vs Control (Δ_Α_ − Δ_C_) p value	ABBI group follow-up (Δ_A2_ = T2 − T0, N = 10)	ABBI-Follow-up vs ABBI (Δ_A_ − Δ_A2_) p value
Auditory localization [deg]	2.80 (1.48)**	1.15 (0.78)	0.0001***	3.83 (2.22)	0.17
Auditory bisection [deg]	2.43 (2.12)***	0.15 (0.66)	0.0005**	3.18 (2.83)	0.11
Auditory distance [deg]	0.88 (2.41)	0.98 (1.66)	0.62	3.06 (1.93)	0.99
Auditory reaching [cm]	25.48 (12.12)***	3.95 (12.19)	0.0005**	17.12 (9.89)	0.17
Proprioceptive reaching [cm]	16.44 (10.40)***	9.89 (7.18)	0.01**	23.78 (17.92)	0.66
General mobility [s]	3.53 (2.59)**	1.94 (0.95)	0.04*	2.97 (2.78)	0.44

One year follow-up of the ABBI group (T2-T0). Data are presented as mean and standard deviation. The stars indicate the statistical significance of the corresponding t-test of the score difference (*p < 0.05; **p < 0.01; ***p < 0.001).

### Pre-post evaluation

#### Auditory localization

The samples resulted normally distributed (KS: Z = 0.603 and Z = 0.802, P > 0.2). The t-test in the ABBI training group between T0 and T1 showed a decrease in threshold (t_(36)_ = 2.85, p = 0.0003), while the same test in the Classical training didn’t result statistically significant. The ANCOVA showed a decrease in threshold between the ABBI training in comparison to the classical training (F_(1,32)_ = 38.29, p = 0.0001, Cohen’s *d* = 1.45, CI = [0.45, 1.81]).

#### Auditory bisection

The samples resulted normally distributed (KS: Z = 0.590 and Z = 0.718, P > 0.2). The t-test in the ABBI training group between T0 and T1 showed a decrease in threshold (t_(36)_ = 4.19, p = 0.0001), while the same test in the Classical training didn’t result statistically significant. The ANCOVA showed a decrease in threshold between the ABBI training in comparison to the classical training (F_(1,32)_ = 14.81, p = 0.0005, Cohen’s *d* = 1.51, CI = [0.51, 1.81]).

#### Auditory distance

The samples resulted normally distributed (KS: Z = 0.992 and Z = 1.207, P > 0.1). Interestingly, the t-test between T0 and T1 didn’t result statistically significant for both the ABBI and Classical training group. In addition, also the ANCOVA didn’t show any statistical difference (F_(1,32)_ = 0.23, p = 0.62).

#### Auditory reaching

The samples resulted normally distributed (KS: Z = 0.436 and Z = 1.067, P > 0.2). The t-test in the ABBI training group between T0 and T1 showed a decrease in threshold (t_(36)_ = 6.47, p = 0.0001), while the same test in the Classical training didn’t result statistically significant. The ANCOVA showed a decrease in threshold between the ABBI training in comparison to the classical training (F_(1,32)_ = 14.71, p = 0.0005, Cohen’s *d* = 1.76, CI = [−4.00, 7.30]).

#### Proprioceptive reaching

The samples resulted normally distributed (KS: Z = 1.371 and Z = 0.787, P > 0.2). The t-test in the ABBI training group between T0 and T1 showed a decrease in threshold (t_(36)_ = 4.18, p = 0.0002), while the same test in the Classical training didn’t result statistically significant. The ANCOVA showed a decrease in threshold between the ABBI training in comparison to the classical training (F_(1,32)_ = 7.47, p = 0.01, Cohen’s *d* = 0.76, CI = [−4.18, 4.08]).

#### General mobility

The samples resulted normally distributed (KS: Z = 1.785 and Z = 1.012, P > 0.2). The t-test in the ABBI training group between T0 and T1 showed a decrease in threshold (t_(36)_ = 1.87, p = 0.03), while the same test in the Classical training didn’t result statistically significant. The ANCOVA showed a decrease in threshold between the ABBI training in comparison to the classical training (F_(1,32)_ = 4.41, p = 0.04, Cohen’s *d* = 0.85, CI = [−0.38, 1.29]).

### One-year follow-up

A long lasting effect of the ABBI training was present. In fact, the two-tailed t-tests within the ABBI training group didn’t show any statistical difference at T1 and T2 for each of the test proposed (p > 0.26). Furthermore, also the Wilconxon test didn’t show any statistical difference between T1-T0 and T2-T0 for each of the test proposed (Z < 1.5, p > 0.12).

## Discussion

We have proposed and tested a new training for enhancing spatial cognition in visually impaired children to strengthen the natural association of auditory and motor signals from the body. Forty-four children aged 6–17 years old participated in the study that provided encouraging results, indicating that the training enhances spatial abilities and the benefits are long lasting. To our knowledge, this is the first study that aims at developing and validating a new sensorimotor technology to rehabilitate spatial perception in the blind child.

The acquisition of spatial skills is particularly important for visually impaired children, and the hearing sense can be used to foster compensatory mechanisms for the development of spatial perception principally because it is the main channel for providing distal information^41^. Multisensory experiences can be more effective than unimodal stimulation in training settings^13,21^ principally because spatial capabilities are acquired during the development thanks to the reciprocal influence between visual perception and execution of movements^28^. From this viewpoint, we developed a multisensory training based on the coupling of motor and auditory signals to provide additional sensory feedback to body movements. Indeed, a recent study from our group showed that a short audio-motor training helps to recalibrate audio spatial perception in visually impaired adults^20^. A similar approach has been used in the present study, to test whether also visually impaired children can benefit from the use of the proposed multisensory training.

We found that the training significantly enhances spatial perception in visually impaired children. Children can experience multisensory audio-motor correspondences that permit to perceive a coherent representation of the surrounding environment. Since the superior colliculus plays an important role in the integration of multiple sensory information that belong to the same event^42^, the positive outcomes of the training might also be mediated by the superior colliculus role in the formation of auditory spatial representation. Indeed, it has been shown that experience with crossmodal signals is fundamental in structuring the multisensory properties of the superior colliculus^43–45^.

Among the tested spatial functions, only the ability to discriminate the relative distance in depth of two sounds doesn’t improve after the proposed training. This might be due to the fact that this ability requires good audio recalibration along the sagittal direction, which is difficult due to the morphology of the human hearing system^46^. A specific audio-motor training, targeting solely this direction, should be probably performed to observe an improvement.

In summary, we demonstrated that the multisensory training proposed has overall positive effects on the development of spatial cognition in visually impaired children.

We consider this approach as innovative for three main reasons. First, visually impaired children don’t need to learn a new language to perform the training because the association of body movement and auditory feedback is natural and relies on engrained processes instinctually learned by the brain without any complex cognitive effort. Several user studies indicated that the most simple, efficient and natural sensory substitution systems, such as the Braille or the cane, are also the most used ones^47^ because they require the active participation of the user and learning occurs unconsciously trough everyday natural training. Our approach is comparable to the idea of natural training because it exploits information that can be naturally decoded by the hearing system. Secondly, the training is based on the important link established by action and perception in the learning process. Experimental results suggests that there is no perception without action^48^. The association between the sensory motor systems for the development of visual-like spatial abilities is a fundamental characteristic of our approach. We use the natural links between action and perception, which are thought to be essential for learning. Thirdly, cognitive strategies of analysis are not required, as our approach needs only limited attentive resources by the user. This is particularly important if considering that visually impaired individuals are often sensory overloaded by the wide amount of acoustical and tactile signals coming from the external environment^49^ while it might be better to convey direct spatial information about body position in space through natural spatial coordinates. Indeed the same approach could be extended to younger children, since low attentional resources are required for the use of the ABBI device.

To conclude, the tailored audio-motor training is a powerful tool to prevent spatial development delays in visually impaired children.

## Data Availability

The datasets generated during the present study are available from the corresponding author on reasonable request.
